# Regulators involved in trophoblast syncytialization in the placenta of intrauterine growth restriction

**DOI:** 10.3389/fendo.2023.1107182

**Published:** 2023-01-31

**Authors:** Hanjing Zhou, Chenqiong Zhao, Peixin Wang, Weijie Yang, Haiyan Zhu, Songying Zhang

**Affiliations:** ^1^ Assisted Reproduction Unit, Department of Obstetrics and Gynecology, Sir Run Run Shaw Hospital, School of Medicine, Zhejiang University, Hangzhou, China; ^2^ Key Laboratory of Reproductive Dysfunction Management of Zhejiang Province, Hangzhou, China

**Keywords:** intrauterine growth restriction, trophoblast, syncytialization, signaling pathway, epigenetic modification, metabolism, senescence, autophagy

## Abstract

Placental dysfunction refers to the insufficiency of placental perfusion and chronic hypoxia during early pregnancy, which impairs placental function and causes inadequate supply of oxygen and nutrients to the fetus, affecting fetal development and health. Fetal intrauterine growth restriction, one of the most common outcomes of pregnancy-induced hypertensions, can be caused by placental dysfunction, resulting from deficient trophoblast syncytialization, inadequate trophoblast invasion and impaired vascular remodeling. During placental development, cytotrophoblasts fuse to form a multinucleated syncytia barrier, which supplies oxygen and nutrients to meet the metabolic demands for fetal growth. A reduction in the cell fusion index and the number of nuclei in the syncytiotrophoblast are found in the placentas of pregnancies complicated by IUGR, suggesting that the occurrence of IUGR may be related to inadequate trophoblast syncytialization. During the multiple processes of trophoblasts syncytialization, specific proteins and several signaling pathways are involved in coordinating these events and regulating placental function. In addition, epigenetic modifications, cell metabolism, senescence, and autophagy are also involved. Study findings have indicated several abnormally expressed syncytialization-related proteins and signaling pathways in the placentas of pregnancies complicated by IUGR, suggesting that these elements may play a crucial role in the occurrence of IUGR. In this review, we discuss the regulators of trophoblast syncytialization and their abnormal expression in the placentas of pregnancies complicated by IUGR.

## Introduction

Small for gestational age (SGA) refers to new-borns whose birth weight is below the 10th percentile for gestational age (1). A pregnancy in which an SGA fetus that cannot reach its genetically determined growth potential at any gestational age is diagnosed with IUGR (2). According to the literature, fetal IUGR occurs in 4% to 7% of global live births each year and occurs either alone or accompanied by pre-eclampsia (PE) or syndrome hemolytic anemia (HELLP) (3, 4). IUGR is the second leading cause of perinatal mortality (5). IUGR not only increases the incidence of fetal distress, premature delivery, and stillbirth, but also significantly increases the risk of various neurological and respiratory diseases in newborns (6, 7). Moreover, the incidence of cognitive impairment in childhood and the risk of heart disease, hypertension, and type 2 diabetes in adulthood are also increased (8–10).

Fetal growth is a continuous process in which cells, tissues, and organs differentiate and undergo maturation. During this process, the transport of primary nutrients, such as glucose, amino acids, and lipids, is essential for the development of a healthy baby (11). The placenta not only maintains the pregnant state and protects the embryo from infection, but also promotes the exchange of nutrients, gases, and waste products so that the embryo can safely survive and grow in a healthy intrauterine environment (12). In pregnancies complicated by placental dysfunction, oxygen and nutrient supply is inadequate to meet the metabolic demand of the growing fetus. This condition may lead to fetal growth restriction and fetal hypoxia, and in severe cases, irreversible ischemic organ damage and intrauterine fetal death may occur (13). Recent study findings suggested that insufficient placental nutrition transport during hypertensive pregnancy impairs fetal growth by decreasing placental protein O-GlcNAcylation (11). The proper functioning of the placenta depends on the integrity of its structure, which involves trophoblasts, immune cells and other placental cells proliferating, differentiating, and undergoing apoptosis at proper rates and in a balanced state. Unlike other placental cells, cytotrophoblasts (CTBs) have a tendency to fuse during pregnancy, which promotes the form of a multinucleated syncytial barrier that constitutes the interface between the maternal and fetal circulation and has the main function of transporting gases, nutrients and wastes between the fetus and the mother. On the other hand, extravillous trophoblasts (EVTs) invade the decidua and arterial vessels to participate in vascular remodeling. In hypertensive pregnancies, dysfunctional placentas showed a deregulation of cell fusion in the formation of the syncytiotrophoblast (STB) and increased apoptosis (14). In addition, the shallow invasion of trophoblasts and incomplete remodeling of the uterine arteries reduced vessel pulsatility, preventing placentas from achieving a steady blood flow to ensure the perfusion of the intervillous space and adequate transit time for exchange (15). In humans, placental tissues from pregnancies complicated by PE showed a lower STB/CTB ratio than normal placentas (16). Similarly, placental tissues from pregnancies complicated by IUGR showed evidence of placental underdevelopment, including villous hypermaturity and distal villous hypoplasia (17). Moreover, primary CTBs isolated from placentas of PE and IUGR pregnancies showed evidence of impaired syncytialization compared to those from normal pregnancies (18). During the multiple processes of trophoblast syncytialization, specific proteins and several signaling pathways are involved in coordinating these events and regulating placental function. It has been reported that cytokines and growth factors act on different signaling pathways to produce a series of cascade effects and to induce the expression of downstream molecules to regulate the syncytialization of trophoblasts (19). In this article, we discuss the relationships between abnormal trophoblast syncytialization and IUGR caused by placental dysfunction.

## The etiology of IUGR

IUGR and PE are two different but clinically relevant pregnancy disorders attributed to an inadequate depth of trophoblast invasion into the maternal endometrium (20). At present, the causes of approximately one-third of IUGR cases including genetic factors, placental dysfunction and maternal influences. The remaining cases of IUGR are classified as idiopathic (21, 22). Although the causes are ambiguous, most of them are frequently associated with placental perfusion insufficiency. In patients with idiopathic IUGR, the placentas are smaller in size, the proliferation of trophoblasts is reduced, and the structures of placental villi are shortened. Interestingly, pathological results show that these small placentas are ischemic, indicating inadequate invasion of trophoblasts into the placental bed and deficient remodeling of the uterine spiral arteries, which eventually leads to chronic placental hypoxia (23, 24).

The development of the placenta depends on the balance of trophoblast proliferation, differentiation, and apoptosis. As with tumor cells, trophoblasts also have the ability to migrate into and invade the endometrium with strict biological regulation (25). On the 12th day after fertilization, CTBs invade STB columns to form primary villi. Approximately 10 days later, the chorionic trophoblasts in contact with maternal decidua differentiate into interstitial cytotrophoblasts (iCTBs) and endovascular cytotrophoblasts (eCTBs). iCTBs then invade the decidual matrix to control the depth of placental implantation and establish contact with decidual matrix cells, giant cells, and uterine natural killer (uNK) cells to accelerate the apoptosis of smooth muscle cells and the degradation of elastin (26–28). The latter passes through arterial walls and replaces vascular endothelial cells to participate in uterine spiral artery remodeling (26). This process changes the state of placental blood vessels from a high-resistance and low-flow state in early pregnancy to a low-resistance and high-volume state in subsequent pregnancy stages, thus improving placental perfusion and promoting villous microvascular formation to ensure sufficient material exchange between the mother and fetus (29). Although the existence of a low oxygen environment in the first trimester of pregnancy is essential to protect the fetus from injury due to excessive oxidative stress, persistently low perfusion with hypoxia can result in the development of pregnancy complications (30). Doppler ultrasound detected increased resistance of uterine spiral arteries in placentas from pregnancies complicated by PE or IUGR (31, 32). Interestingly, the clinical outcomes depend on the number of arteries involved and the extent of arterial involvement in remodeling in these pregnancies (33, 34).

To date, most scholars believe that the reduced migration and survivability of trophoblasts may be a key feature leading to IUGR. However, changes in other trophoblast cell behaviors can also lead to IUGR. In early pregnancy, placental CTBs proliferate and fuse to form a multinucleated syncytial barrier that mediates immune tolerance, steroid and peptide hormone synthesis, nutrient and gas exchange, and waste product removal between the mother and fetus. Apoptosis occurs throughout placental development, which causes senescent trophoblasts or damaged syncytia to be continuously released into the maternal circulation in the form of fragments or vesicles; this process is called syncytial deportation (35). In normal placentas, the formation and deportation of the syncytial barrier are in equilibrium. Nevertheless, as fetal growth is closely correlated with the nutrient supply mediated by the syncytium, the imbalance between trophoblast syncytialization and syncytial deportation could lead to nutrient deficiency and eventually cause pathologies such as PE, fetal IUGR, and embryonic death (36, 37). It has been reported that placentas from patients with pregnancies complicated by IUGR showed a reduced fusion index, which was calculated as “the ratio of the number of nuclei in the syncytia divided by the total number of nuclei”, where the syncytium was defined as at least three nuclei surrounded by a cell membrane (38, 39). In addition, studies have also shown that the proportion of syncytial deportation to the maternal circulation increased, while the expression of the fusion protein syncytin-1, which mediates syncytia formation, was reduced in placentas from pregnancies complicated by PE and IUGR (35). Furthermore, cultured CTBs from pregnancies complicated by PE and HELLP-associated IUGR were correlated with a pronounced lower cell fusion index, human chorionic gonadotropin beta (β-hCG) secretion, *syncytin* gene expression, and a significantly higher apoptosis rate (14). The above findings suggest that the occurrence of IUGR may be related to insufficient syncytialization of trophoblasts (14, 40).

## Trophoblast syncytialization

**Figure 1:**
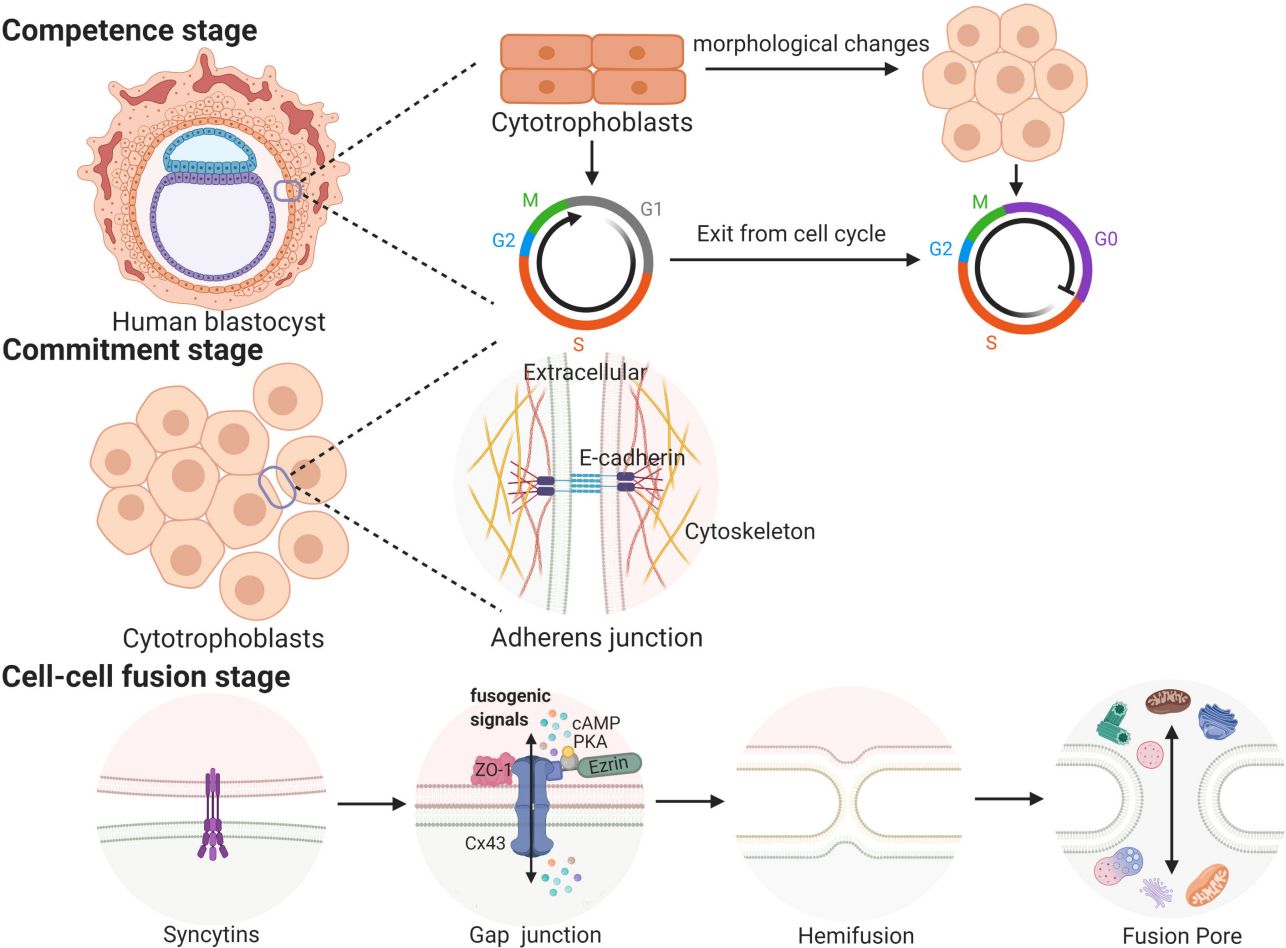
The three stages of trophoblast syncytialization.

Cell fusion processes occurring in a variety of biological contexts share many steps that are tightly regulated in space and time. Trophoblast cell fusion is mainly divided into three steps, in which a variety of proteins are involved and function in a space- and time-regulated manner (41, 42). For example, the first stage (competence stage) involves cell morphological changes with proliferative activity loss. The second stage (commitment stage) is characterized by cell adhesion and communication processes that lead to the activation, expression, exposure or assembly of the fusogenic machinery. In this stage, adherens junctions, tight junctions and gap junctions trigger the commitment of primary cells, followed by their fusion (39). The final stage (cell-cell fusion stage) is defined by the merging of two plasma membranes and the mixing of cellular contents (43) (see **Figure 1**).

In placentas, the process in which CTBs break down cell boundaries, and gradually fuse into a multinucleated STB layer is called trophoblast syncytialization (44). Under the physiological conditions of pregnancy, syncytialization is divided into two stages. The first stage begins on the 7th day and lasts until the 11th day after fertilization. During this phase, trophoblasts make contact with the maternal endometrium and begin to differentiate into STB. Then, STB fuse and lose their cell boundaries to form a multinucleated structure called the primitive syncytium, which facilitates the implantation of the embryo into the maternal endometrium (39, 45). After blastocysts are completely implanted, the fusion between STB ceases, while the proliferation of CTBs continues (39). The second stage begins on the 12th day after fertilization. As the pregnancy progresses, the surface area of the villi is expanded and regenerated, which depends on the continuous fusion of CTBs. This stage continues until delivery (45). These two syncytialization stages involve two different types of trophoblasts, and the processes involved in these two stages may be regulated by different mechanisms (45). The first stage of syncytialization occurs after early apoptosis. A series of apoptotic cascades cause negatively charged phosphatidylserines to accumulate in the outside of the cell membrane, which is a prerequisite for the fusion of trophoblasts (46, 47). However, it is not clear whether these processes of apoptosis or differentiation are the same prior to cell fusion because the initial stages of these two processes depend on the same molecular mechanism (48, 49). During the second stage of syncytialization, when the CTBs begin to fuse into the STB layer, the apoptotic cascade is immediately suppressed by high levels of apoptosis suppressors, such as MCL-1 and BCL-2. After the fusion is completed, the apoptotic cascade is restarted; this induces syncytial knots to be released into the maternal circulation (48). The result of syncytialization is the creation of a complete epithelial-like barrier covering the surface of all villi. The STB layer is supplied by the mother’s blood and transports oxygen and nutrients to the developing fetus. As a barrier between the mother and the fetus, the STB layer protects the fetus from attack by pathogens and the mother’s immune system. In addition, the STB can also secrete a large number of hormones, including β-hCG; the presence of this hormone provides the basis of one of the diagnostic criteria used to confirm early pregnancy (45).

## Syncytialization-related proteins

A multitude of proteins are strictly regulated to participate in cell fusion *via* appropriate mechanisms at the correct place at the right time [reviewed in (39)]. For example, the first stage involves hCG, which has the capacity to induce cell differentiation and the formation of the syncytia. The second stage involves certain gap junction proteins, including connexins, that enhance cellular communication by forming intercellular channels (50). In addition, the expression levels of several cell adhesion molecules, including cadherins and zonula occludens-1 (ZO-1), are altered during the trophoblast syncytialization process. In the final stage, fusogenic proteins (syncytin-1 and syncytin-2), along with their transcription factor (glial cell missing 1 (GCM1)), play a crucial role in trophoblast syncytialization (51). Furthermore, 11β-hydroxysteroid dehydrogenase 2 (11β-HSD2) can convert cortisol into inactive metabolites and is also regarded as a biochemical marker of syncytialization (52).

### hCG

During the early stages of pregnancy, hCG is secreted by embryonic trophoblasts. In contrast, during the latter phases of pregnancy, hCG is produced by the STB layer (39). hCG is a heterodimer composed of α and β subunits and serves as the trigger for the formation of the primitive syncytium; it also promotes syncytialization *via* an autoparacrine loop (53, 54). Research has shown that hCG is vital for the production of estrogen and preventing the decline in progesterone as a result of the degradation of the corpus luteum during early pregnancy (55, 56). hCG is also involved in promoting trophoblast invasion; regulating the growth of the uterus, fetus, and placenta; and protecting the fetus from attack by the mother’s immune system (56, 57). β-hCG is expressed only in the STB layer, and the level rises gradually with cell fusion; consequently, hCG is regarded as a biochemical marker of trophoblast syncytialization (58, 59). Typically, hCG increases intracellular cAMP levels *via* autocrine methods and regulates the expression of both GCM1 and syncytin-1 through the cAMP/PKA pathway to promote the syncytialization of trophoblasts (60). In addition, the abnormally low or high maternal serum levels of free β-hCG during different trimesters are closely associated with adverse pregnancy outcomes, including fetal IUGR, spontaneous abortion, and preterm birth. For this reason, free β-hCG is also regarded as a predictor of pregnancy complications (61, 62).

### Cadherins

Cadherins are calcium-dependent integral member glycoproteins that not only function as cell shape and polarity stabilizers by promoting the formation of adherens junctions but also contribute to the balance of cell proliferation, migration and tissue homeostasis by interacting with the intracellular cytoskeleton (63). Cell-cell adhesion is triggered by the extracellular N-terminal domain of cadherins clustering with cadherins located on neighboring cells. E-cadherin, localized in the cell membrane of epithelial trophoblasts (cell columns and CTBs) and the inner STB, is known to be decreased after cell fusion, and is regarded as a morphological marker of cell fusion (16, 64, 65). Specific antibodies against the extracellular domain of E-cadherin have been shown to impede the human trophoblast syncytialization process by disrupting the aggregation of mononuclear CTBs, indicating that E-cadherin is directly involved in the cellular adhesion step of trophoblast syncytialization (66). It has been reported that E-cadherin is involved in regulating human trophoblast syncytialization by interacting with the β-catenin signaling complex and mediating the formation of cell junctions (67). In contrast to E-cadherin expression, cadherin-11 expression increases during trophoblast syncytialization (68). Cadherin-11 antisense treatment resulted in cellular aggregation but fusion deficiency in human trophoblasts. These data suggest that cadherins are vital for the whole process of trophoblast syncytialization, in which E-cadherin mediates mononuclear cell aggregation, while cadherin-11 is required for syncytialization.

Successful placentation relies on appropriate formation of syncytia and homing of trophoblasts to maternal spiral arteries. These processes involve a number of cell-cell adhesion molecules, the abnormal expression of which ultimately results in impaired placentation (69). In the placentas of pregnancies complicated by PE and IUGR, abnormally elevated cadherin levels may participate in the destruction of epithelial-mesenchymal transition (EMT) and the alteration of epithelial/mesenchymal balance, finally resulting in a shallower depth of trophoblast invasion into the decidua (20). Although cadherins are required for the entire trophoblast syncytialization process and change dynamically in the placentas of pregnancies complicated by IUGR and PE, until recently, there was a lack of direct evidence of abnormal cadherin expression causing fetal IUGR owing to syncytialization deficiency.

### ZO-1

Tight junctions consist of transmembrane proteins such as the cytoplasmic scaffolding protein ZO-1 that regulate cell-cell adhesion and contribute to epithelial barrier function (70). ZO-1 is a 220 kDa protein that zips cells together and maintains cell polarity. In a mouse model, *ZO-1* knockout (KO) induced defects in mouse placental development, mainly in vascular tree formation and chorioallantoic fusion (71). In humans, the involvement of ZO-1 in cell fusion and subsequent trophoblast differentiation has been established by morphological and biochemical data. ZO-1 is localized mainly in CTBs and at the intercellular boundaries between CTBs and between CTBs and STB, where its expression substantially decreases during cell fusion (72). In human primary trophoblast cultures, ZO-1 was predominant during the aggregation of CTBs and then decreased drastically with cell fusion. It has been proven that the decrease in ZO-1 induces cell fusion by establishing gap junction communication between two fusion-competent cells, where the expression of the gap junction protein connexin 43 is upregulated to initiate cell fusion (39, 72). In PE placentas, the expression levels of E-cadherin and ZO-1 were elevated compared to the controls (73). MiR-200 family members are highly relevant to PE and IUGR pregnancies. MiR-200 family impaired trophoblast invasion and altered the EMT process by stimulating the expression of the epithelial markers E-cadherin and ZO-1 (74). In contrast, Misan et al. reported that ZO-1 levels in both serum and placentas showed no significant difference between IUGR and control groups (75). Although recent study findings suggest that ZO-1 participates in the trophoblast syncytialization process, there is not sufficient evidence to prove that the occurrence of IUGR is related to the syncytialization deficiency caused by abnormal expression of ZO-1, which may be a new direction for future research.

### Syncytins

Syncytins are endogenous retroviral envelope proteins containing a disulfide sequence, a furin cleavage site, a fusion peptide, and a receptor-binding domain. There are two pairs of retrovirus-derived envelope genes named *syncytin-1* and *syncytin-2* in humans and *syncytin-A* and *syncytin-B* in mice (76, 77). Both are expressed in placental trophoblasts and specifically mediate the formation of the STB layer, particularly through their fusogenic activity (78–80). In humans, syncytin-1 mediates cell fusion first by seeding its fusion peptide into the targeting membrane, then bending the cytomembrane, and finally forming fusion pores (39). During the spontaneous syncytialization process of human primary CTBs, the expression level of syncytin-1 is increased (81). It has been reported that the silencing of *syncytin-1* gene expression could significantly reduce β-hCG secretion and cell fusion (7, 51). In contrast, after transferring syncytin-1 vectors into BeWo choriocarcinoma cell lines without forskolin induction, cell-cell fusion was directly induced, suggesting that syncytin-1 may be directly involved in regulating the syncytialization of trophoblasts (51, 78). Syncytin-2 is another fusion protein expressed only in CTBs, and its receptor, major facilitator superfamily domain-containing 2 (MFSD2), is also found in CTBs (82). A previous study showed that syncytin-2 was confined to G0 cells when all trophoblasts were ready to fuse, and the overexpression of syncytin-2 resulted in the unstable fusion of cells in their S/G2/M phases (83). As syncytin-2 works on CTBs and induces the initiation of cell fusion, syncytin-2 could be regarded as a marker of the initiation of trophoblast syncytialization (84). Reduced expression of syncytin-1 and syncytin-2 was detected in IUGR placentas compared to controls (14, 38). In mice, syncytin-A disruption caused fetal IUGR and embryonic lethality by reducing glucose transport between the maternal-fetal interface (85). *Syncytin-A* gene KO changed placental morphology, resulting in low expression of neovascularization-related genes and widespread vascular abnormalities in the labyrinth, which were characterized by irregular distribution and reduced numbers of fetal vessels (86). In addition, *BCL9L*-deficient mice exhibited a striking downregulation of syncytin-A in the placenta with severe disruption of trophoblast fusion (87, 88). Moreover, *syncytin-A* null mouse embryos died between embryonic days E11.5 and E13.5 due to the failure of placental formation (89). The level of the human antiangiogenic molecule sFlt-1 was markedly increased in *syncytin-A* KO mice, which prevented spongiotrophoblast from differentiating into glycogen cells and reduced the exchange area of the labyrinth and glycogen stores, which were highly relevant to fetal IUGR (86, 90). Therefore, syncytins directly contribute to trophoblast syncytialization, and placental syncytin deficiency may be associated with the occurrence and development of fetal IUGR in both humans and mice (38).

### GCM1

GCM1 is a key transcription factor that regulates placental development and is predominantly expressed in mammalian trophoblasts, regulating cell differentiation, turnover and maintenance (91, 92). GCM1 functions as a regulator of STB formation and the expression of fusogenic genes such as *syncytin-1* and *syncytin-2* (93, 94). GCM1 regulates *syncytin* gene expression by binding to two GCM1-binding sites located upstream of the 5’-long terminal repeats of the *syncytin-1* promoter region, which is essential for cell fusion (93, 95, 96). A previous study found that syncytin-A was downregulated in the placenta of *GCM1*-deficient mice (97). In humans, both reduced and increased levels of GCM1 have been described in several pregnancy complications and have been linked with altered trophoblast function *in vitro* (98–100). The expression level and transcriptional activity of GCM1 are reduced in the primary CTBs of first trimester and term pregnancies under hypoxia, along with syncytia formation deficiency (87, 101, 102). In addition, the excessive expression of syncytin-1 caused by abnormal regulation of GCM1 also leads to extensive cell fusion and cell death (103). Moreover, GCM1 promotes the transcription of syncytin-2 following interaction with the cell cycle inhibitor p21 (83). It has been reported that GCM1 is among the top scoring genes with the greatest negative association with fetal growth in human placentas from pregnancies complicated by IUGR (104). The transcription factor p45 NF-E2 (nuclear factor erythroid derived 2) has recently been found to regulate trophoblast differentiation, and its absence causes placental insufficiency and IUGR in mice (105). P45 NF-E2 negatively regulates human STB differentiation and apoptosis activation by modulating GCM1 acetylation and sumoylation, which is associated with IUGR (92). Collectively, these results suggest that the transcription factor GCM1 may play a crucial role in the trophoblast syncytialization process and that its abnormal regulation may lead to the occurrence of IUGR caused by syncytialization deficiency.

### 11β-HSD2

11β-HSD2 is an NAD^+^-dependent oxidase that converts active cortisol to inactive cortisone and is expressed from the earliest 3 weeks after embryo implantation. The levels of 11β-HSD2 drop intensely during the third trimester. 11β-HSD2 localizes in the STB layer, where it acts as a placental glucocorticoid barrier to protect the fetus from excessive maternal glucocorticoid disturbance (106–108). It is reported hCG upsurges 11β-HSD2 expression by activating the cAMP/PKA pathway, resulting in histones modification alteration and specificity protein 1 (Sp1) expression increase, which activates the transcription of *HSD11B2* during trophoblast syncytialization (109). In IUGR pregnancy caused by different etiologies, placental 11β‐HSD2 expression is attenuated by distinct mechanisms. For examples, stress and nutritional deprivation reduce 11β‐HSD2 expression by increasing its methylation, while hypoxia decrease 11β‐HSD2 expression *via* alternative mechanisms rather than by methylation. A recent study revealed that the accumulation of cadmium in the placenta causes fetal IUGR by downregulating 11β‐HSD2 expression *via* Sp1, which binds to GC‐rich sections of the *11β‐HSD2* promoter region (110, 111; ). Although most of studies have shown the reduced level and activity of placental 11β-HSD2 in pregnancies complicated by PE and fetal IUGR, and the impairment of 11β‐HSD2 glucocorticoid barrier is associated with fetal IUGR and the development of chronic diseases in later life (109, 112, 113). Nevertheless, increased maternal 11β-HSD2 activity was observed many weeks before the clinical manifestations of PE and preterm fetal IUGR appeared (114).

## The signaling pathways involved in syncytialization

**Figure 2:**
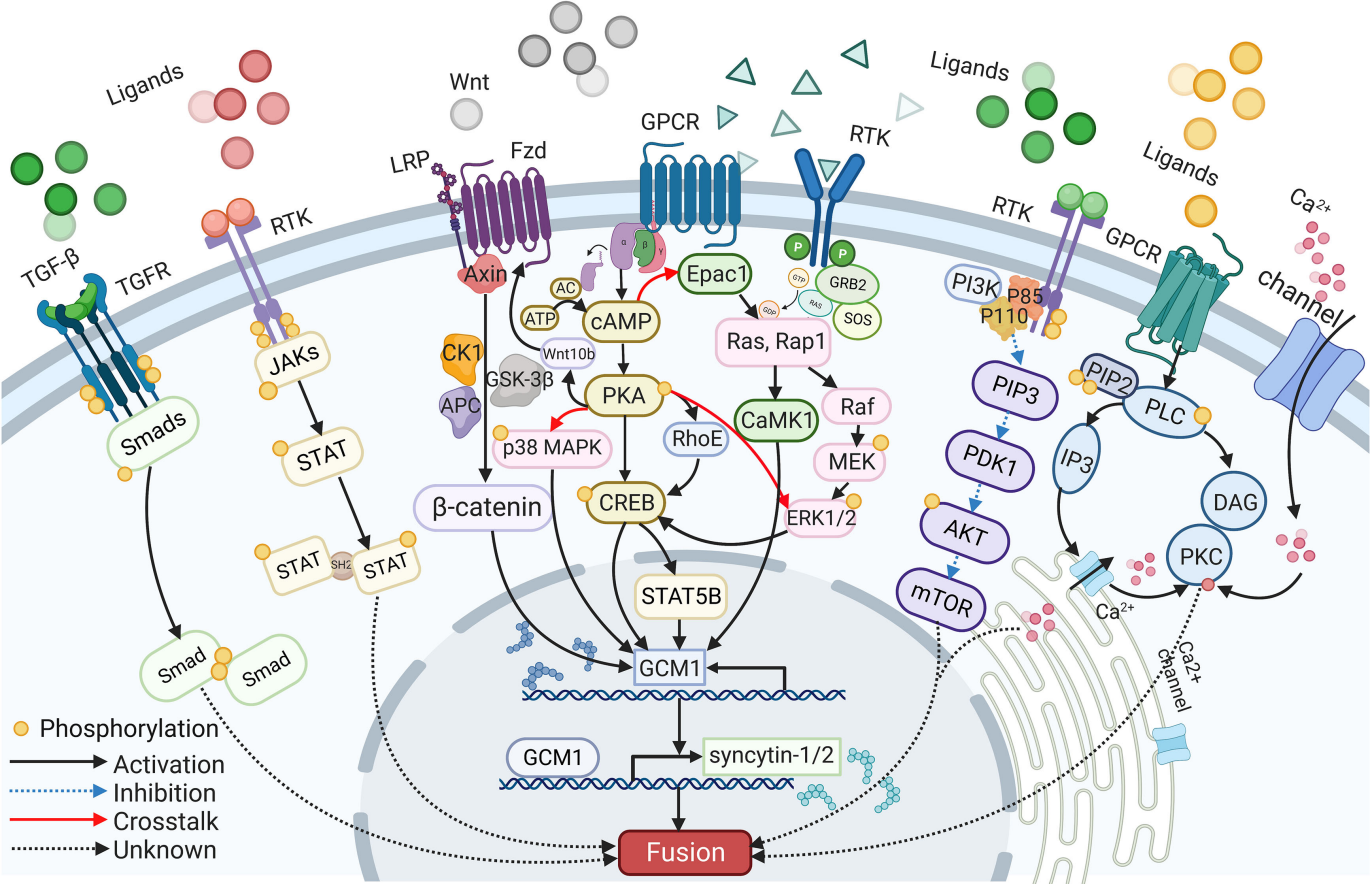
The signaling pathways participate in syncytialization.

A wide range of intracellular molecules are known to participate in the regulation of trophoblast syncytialization. *In vitro*, research has identified several signaling pathways, including the cAMP/PKA, Wnt/β-Catenin, MAPK, PI3K/AKT, JAK/STAT, and TGF-β/SMAD signaling pathways, that regulate trophoblast syncytialization by targeting syncytialization-related proteins or in other ways (see **Figure 2**). Furthermore, abnormalities in these signaling pathways have also been reported in cases of fetal IUGR caused by placental dysfunction.

### cAMP dependent signaling pathways

The cAMP signaling pathway is one of the most common signaling pathways involved in cell fusion; this pathway is strictly controlled by a range of regulators that act in a spatial and temporal manner to convey appropriate messages (115). Signaling molecules, such as hCG and forskolin, first act on their receptors to activate adenylate cyclase (AC). This, combined with ATP catalysis, results in an increase in the intracellular level of cAMP (115). As a second messenger, cAMP then activates various downstream intracellular molecules, such as cAMP-dependent protein kinase (PKA) and the exchange protein that is directly activated by cAMP1 (EPAC1) (116, 117). Both of these factors eventually target fusogenic genes, leading to cell fusion.

PKA mediates many cAMP-induced biological effects, including cell fusion (19). For example, the activation of the cAMP/PKA signaling pathway by forskolin and β-hCG has a positive impact on trophoblast fusion (60, 118). Furthermore, the inhibition of fusion arising from hypoxia can be alleviated through the activation of the PKA pathway (119). Increased levels of fusion were detected in BeWo cells following the transfer of PKA plasmids; this was associated with upregulated transcriptional activity of GCM1 and an increase in the levels of syncytin-1 protein. These studies also showed that fusion was compromised when a PKA inhibitor, such as H89, was added (120, 121). Moreover, the expression levels and distribution of type I and type II PKA showed changes during the fusion between human CTBs; this was considered to be associated with the secretion of hormones and the reorganization of the cytoskeleton (120). Research has also proven that activated cAMP/PKA phosphorylates CREB (cAMP response element-binding protein), a downstream transcription factor that binds to CBP (CREB binding protein) and P300, to increase the expression levels of several fusogenic genes, including *syncytins*, *hCG*, *GCM1*, and *Cx43* (39). Furthermore, cAMP, PKA, and CREB upregulate GCM1, then increase the expression levels of syncytin-2 *via* STAT5B (84). Furthermore, previous research found that cAMP can also upregulate the expression levels of the GTP-binding protein RhoE *via* cAMP and PKA, while RhoE was shown to influence the fusion of BeWo cells by activating the transcription factor GCM1 (122).

It is reported that ethanol reduces placental 11β-HSD2 expression *via* cAMP/PKA signaling, thus leads to glucocorticoids over-exposure for fetuses, which eventually induces fetal IUGR (123). Moreover, caffeine reduces placental 11β-HSD2 by decreasing intracellular level of cAMP, which is linked to fetal IUGR (124).

### Wnt/β-Catenin signaling pathways

Research has shown that Wnt family members play a key role in embryonic development and tumorigenesis (125). In human cells, Wnt signaling pathways include the classic Wnt/β-catenin signaling pathway, the nonclassic Wnt/Ca^2+^ signaling pathway, and the nonclassic cell polarity pathway (126). The activation of the classic Wnt signaling pathway depends on the binding of Wnt ligands to the heterodimeric frizzled protein (FZD) or the low-density lipoprotein receptor-related protein (LRP-5/6) receptor on the cytomembrane, thereby destroying the CK1-GSK-3β-AXIN-APC phosphorylation complex; this prevents β-catenin from being degraded after undergoing phosphorylation in the cytoplasm and entering the nucleus to bind to transcription factors such as T-cell factor 4 (TCF4) and lymphocyte enhanced binding factor (LEF). Finally, target genes such as *c-Myc*, *Cyclin D1*, and *Mmp7* are activated, which causes abnormal cell proliferation and apoptosis (25).

The classic Wnt/β-Catenin signaling pathway is associated with trophoblast syncytialization. *In vitro*, silencing of TCF-4 or β-Catenin has been shown to inhibit forskolin-induced BeWo cell fusion, at least to a certain extent (88). Other studies found that GCM1 upregulates FZD5 and that elevated levels of FZD5, in combination with nuclear β-Catenin signaling, can maintain the expression of GCM1 during trophoblast differentiation and chorionic branching morphogenesis. These results indicated that Wnt/β-Catenin regulates the syncytialization of trophoblasts by directly targeting GCM1 (127). The expression levels of Wnt10b have also been shown to increase in BeWo cells during the process of forskolin-induced fusion (128). In contrast, the protein levels of Wnt10b, and the nuclear concentration of β-Catenin were both found to be decreased after the addition of a PKA inhibitor. This indicated that cAMP may upregulate Wnt10b *via* the PKA pathway. Wnt10b promoted the migration of β-Catenin into the nucleus by activating the classic Wnt/β-Catenin signaling pathway, which acts on GCM1 and finally upregulates the expression of syncytin-1 (121).

Dickkopf1 (DKK1) is a secreted glycoprotein that can block the classic Wnt/β-Catenin signaling pathway by binding to LRP5/6 (129). A previous study found that the overexpression of the transcription factor HOXB7 inhibited the differentiation of human trophoblasts by downregulating the expression of DKK1 and the transcription of Wnt1/β-Catenin in the placentas of pregnancies complicated by IUGR. This indicated that the Wnt/β-Catenin signaling pathway may play a significant role in the pathogenesis of IUGR (130). Secreted frizzled-related protein (SFRP) is another Wnt signaling pathway inhibitor. In rats, the increased expression of SFRP4 and the reduced expression of nuclear β-Catenin were related to reduced growth in certain regions of the placenta after glucocorticoid-induced growth restriction (131). Furthermore, SFRP1 and SFRP3 were reported to be highly expressed in human placentas from pregnancies complicated by IUGR (132–134).

### The MAPK signaling pathway

The MAPK signaling pathway plays an important role in cell proliferation, differentiation, invasion, aging, and apoptosis (135–138). When signaling molecules act on their receptors, the signal is transmitted to the nucleus by activating a series of factors within the cascade, including MAPKKK, MAPKK, and MAPK, thereby regulating the activity of transcription factors and the expression of target genes; collectively, these actions induce a range of intracellular responses (125). The MAPK family includes four different kinases: extracellular regulated kinase (ERKs), ERK5, c-JUN N-terminal kinase (JNKs), and p38 MAPK (125, 135). ERKs are mainly activated through mitogenic signals, while JNKs and p38 MAPK respond to stress and inflammatory conditions (139).

The ERK1/2 and p38 MAPK signaling pathways regulate the differentiation and fusion of trophoblasts under the effect of various growth factors and cytokines. For example, epidermal growth factor (EGF) and leukemia inhibitory factor (LIF) both exert a positive effect on the syncytialization of trophoblasts *via* MAPK signaling pathways, while specific inhibitors of MAPKs have an opposite effect on primary trophoblast cultures (140–142). Some data have shown that the MAPK and cAMP/PKA signaling pathways communicate by crosstalk. It has been reported that ERK1/2 can be phosphorylated by either PKA or RAP1, factors that are activated by members of the Ras superfamily (143, 144). Conversely, CREB can also be activated by ERK1/2. Together, these two signaling pathways regulate the expression of fusogenic genes, such as syncytin-1 through GCM1 and collectively mediate cell fusion (145). Furthermore, cAMP/PKA and p38α can both promote the expression of syncytin-1 and hCG *via* PPARγ/RXRα during the fusion of BeWo cells and primary human trophoblasts (146).

Placental dysfunction could lead to the development of fetal IUGR; these cases were characterized by the significantly reduced expression of p38 MAPK (147). For example, the expression of both p38 and syncytin-1 was both found to be downregulated in placentas from pregnancies complicated by IUGR, while levels of the transcription factor PPARγ/RXRα remained unchanged (146). Previous research found that insulin-like growth factors (IGFs) promoted trophoblast syncytialization and proliferation *in vitro* by binding to their receptor (IGF-IR) *via* the MAPK signaling pathway (148). Moreover, marked impairment of the coordinated activation of MAPKs, with reduced p38 and JNK phosphorylation, was observed in placentas from pregnancies complicated by IUGR; these factors resulted in extensive placental apoptosis and the impairment of maternal-fetal exchange functions (149).

### The PI3K/AKT signaling pathway

The PI3K/AKT signaling pathway is of great significance for regulating trophoblast proliferation, differentiation, migration, and invasion (150, 151). PI3K is a heterodimer composed of the p85 regulatory subunit and p110 catalytic subunit and has both lipid kinase activity and protein kinase activity (152, 153). When signaling molecules such as hormones and growth factors bind to their receptors on the cell surface, PI3K is activated. Then, activated PI3K phosphorylates phosphatidylinositol-4,5-bisphosphate (PIP2) to phosphatidylinositol-3,4,5-triphosphate (PIP3) (154). Subsequently, AKT is recruited and activated by PIP3 with the help of PDK1, and activated AKT then phosphorylates downstream factors such as mammalian rapamycin (mTOR) to transduce signaling messages (155).

Studies have found that in addition to increasing cAMP levels, simultaneous inhibition of the PI3K/AKT signaling pathway and reduction of intracellular calcium also result in BeWo cell fusion. Moreover, individual blockade of calcium channel function or PI3K/AKT signaling potentiate cell fusion combined with forskolin (156). The above finding suggests that the PI3K/AKT pathway may be involved in the process of trophoblast syncytialization with cAMP activation, but the mechanism of its effects on syncytialization is still unclear.

Researchers have found that some components of the PI3K/AKT signaling pathway are decreased in dysfunctional human placentas, which causes fetal IUGR. For example, defects in p110α signaling impair angiogenesis, leading to placental regional morphogenesis alteration and placental exchange deficiency, which is associated with a severe and early-onset form of IUGR (157–159). *Akt-1* KO mice have fetal growth restriction due to placental insufficiency (160). In human placentas of pregnancies complicated by IUGR, the expression level and activity of mTOR and its upstream molecule AKT are reduced, while those of AMPKα, a negative regulator of mTOR, are increased (161).

### The JAK/STAT signaling pathway

Cytokines and growth factors interact with RTK on the cell membrane to activate JAK. Activated JAK subsequently phosphorylates tyrosine residues of STAT, and the latter forms dimers or multimers through its SH2 domain and is transported from the cytoplasm to the nucleus, where it combines with DNA sequences to activate gene transcription, leading to cell proliferation, differentiation, migration, and apoptosis (162–166).

A significant increase in STAT3 expression was observed during the process of forskolin-induced syncytialization of BeWo cells, and the spontaneous differentiation of primary trophoblasts was also associated with an increase in STAT3 expression (167). The above results suggest that STAT3 may participate in trophoblast syncytialization *in vitro*. In addition, the JAK/STAT signaling pathway also participates in BeWo cell fusion and β-hCG secretion mediated by LIF (142).

The study found that the expression of p-STAT3, a key molecule in the JAK/STAT signaling pathway, was decreased in human placentas from pregnancies complicated by IUGR. In addition, the expression of the JAK/STAT signaling pathway target genes *IFNAR1* and *IFNAR2* was also significantly downregulated in human IUGR placentas, suggesting that the JAK/STAT signaling pathway was inhibited (168). Moreover, the mRNA level of STAT5B is also decreased in the STB of placentas from pregnancies complicated by IUGR, which may affect syncytin-2 expression through GCM1, resulting in insufficient cell fusion (84, 167). STAT3 is located on the STB layer. The decreased level of STAT3 may lead to premature differentiation and increased apoptosis or shedding of STB (48).

### The TGF-β/SMAD signaling pathway

TGF-β is a member of the transforming growth factor family, which is important for cell proliferation, differentiation, migration, apoptosis, and extracellular matrix deposition (169). TGF-β transmits signals through SMAD-dependent and non-SMAD-dependent pathways (170–172). For the SMAD-dependent pathway, TGF-β activates its type II receptors and recruits and phosphorylates type I receptors, and the activated dimeric receptor complex in turn activates SMAD transcription factors and induces them to enter the nucleus to regulate target gene transcription (173).

Expressed by STB, TGF-β negatively controls the fusion of CTBs into syncytia (174). A study revealed that after adding TGF-β1 to differentiated human primary trophoblasts, the potential for syncytialization was decreased with a reduction in hCG and human placental prolactin (hPL) secretion, suggesting that TGF-β1 signaling pathway may affect trophoblast syncytialization by generating a negative effect on the differentiation of trophoblasts, but the specific mechanism is not yet clear (175).

Aberrant activation of the TGF-β signaling pathway causes abnormal development of the placenta and induces disorders such as pregnancy-induced hypertension (PIH) (176, 177). It has been reported that TGF-β1 affects trophoblast invasion and migration abilities by suppressing EMT progression, which disrupts placental vascular remodeling, eventually inducing the occurrence and development of PE. At present, most studies have confirmed that the TGF-β1/SMAD signaling pathway is involved in the development of PE, and it is an integral part of PE treatment. For example, placenta-derived peptide regulates placental function during PE progression *via* the TGF-β1/SMAD signaling pathway (178). In addition, miR-140-5p may be involved in PIH progression by regulating the TGF-β1/SMAD signaling pathway (179). In IUGR placenta, the abnormal TGF-β signaling leads to dysregulated sphingolipoid metabolism, which may favor increased trophoblast cell death (180).

Although these signaling pathways are involved in the regulation of trophoblast syncytialization and the components of which are abnormally expressed in placentas from pregnancies complicated by IUGR, there is still a lack of direct evidence to prove that the aberrant expression of these signaling pathways leads to IUGR by impairing trophoblast syncytialization, which may be a promising direction for further research.

## Epigenetic modifications involved in syncytialization

The *syncytin-1* gene contains two long terminal repeat (LTR) regions: the 5’LTR and the 3’LTR. The U3 region of the 5’LTR overlaps with the CpG island, which extends from the proximal promoter region to the first exon, and the tissue-specific expression of *syncytin-1* is determined by the degree of DNA methylation (181). Studies have found a negative relationship between *syncytin-1* expression and gene methylation. Previous studies of both placentas from pregnancies complicated by PE and placentas from pregnancies complicated by IUGR reported the downregulation of *syncytin-1* caused by promoter hypermethylation resulting from the overexpression of DNA methyltransferase (14, 182b; 183). However, in another study, Makaroun et al. detected a marked increase in *syncytin-1* expression accompanied by reduced levels of *syncytin-1* and *syncytin-2* methylation in placentas from pregnancies complicated by IUGR (184). This concurred with the findings of Gao et al., who reported that the expression of *syncytin-1* was upregulated due to insufficient promoter methylation in the placentas of pregnancies with discordant twins that were SGA (185). One speculation is that this may represent a compensatory mechanism for fetal growth retardation caused by placental dysfunction (186). The administration of 5-AZA-2’deoxycytidine (5-AZA), a DNA demethylation agent, into a range of trophoblast-like cell lines during their fusion process led to a reduction in 5’LTR methylation and an increase in the expression levels of syncytin-1 and hCG (183).

## GCM1 ubiquitination, acetylation, and sumoylation

The ubiquitin-proteasome degradation system plays an essential role in many cellular processes, including cell cycle progression, signal transduction, transcriptional regulation, receptor downregulation, and endocytosis. Studies have found that the human GCM1 (HGCM1) protein has poor stability and is degraded by the ubiquitin-proteasome degradation system under the influence of SCF-human F box protein FBW2 (hFBW2)-E3 (SCFhFBW2E3) complex ubiquitination during cell fusion, thereby permitting GCM1 to be regulated on the posttranslational level (187). Given that GCM1 regulates syncytin-1-mediated trophoblast fusion, Yang et al. speculated that the abnormal expression of hFBW2 may hinder placental development (187). In addition, the activated cAMP protects GCM1 from being degraded by FBW2-mediated ubiquitination *via* two independent pathways: the cAMP/PKA pathway or the cAMP/EPAC1/CaMK1 pathway (60, 188). Studies have already demonstrated reduced levels of HGCM1 protein in the placentas of patients with PE (189). Hypoxia is the leading cause of PE and is known to activate and recruit GSK-3β and FBW2, respectively, to trigger ubiquitination and the degradation of GCM1 *via* the PI3K/AKT signaling pathway (190). During the fusion of placental trophoblasts, histone acetyltransferases (HATs) and histone deacetylases (HDACs) play a joint role in regulating the degree of GCM1 acetylation and thus determine the transcriptional activity of GCM1 (191). It has been reported that the cAMP/PKA signaling pathway recruits CBP to mediate the acetylation of GCM1 following phosphorylation at the Ser269 and Ser275 sites (60). Moreover, the cAMP/EPAC1/CaMK1 pathway subsequently enhances the binding of GCM1 to its target genes by increasing GCM1 desumoylation (192). Collectively, these actions eventually promote cell fusion by targeting the genes downstream of GCM1, such as *syncytins*. Furthermore, increased acetylation and desumoylation of GCM1 have been observed in the placentas from pregnancies complicated by IUGR, thus indicating that the posttranslational modification of GCM1 may be involved in the occurrence of fetal IUGR (92).

## Metabolism and syncytialization

### Hypoxia

In the early stages of embryonic development, the uterus is a hypoxic environment. It continues until the completion of vascular remodeling and the fetal-maternal interface becomes full of blood from the mother, thus providing oxygen to the developing fetus. If vascular remodeling fails, then there is a direct influence on the fetus with respect to the source of oxygen. The failure of vascular modeling can also damage the structure and function of the placenta *via* a range of different mechanisms; this can also have indirect effects on fetal development, thus leading to IUGR.

CTBs spontaneously fused into multinucleated STB in an environment where the concentration of oxygen is 21%; when the oxygen level was reduced to 10%, the majority of trophoblasts remained mononucleated, and there was a significant reduction in the secretion of hCG and hPL (193). Research has shown that under hypoxic conditions (the concentration of oxygen is 1%), there is a reduction in the expression levels of GCM1 and syncytin-1 in primary human trophoblasts, along with reduced levels of cell fusion (87). Hypoxia-inducible factor (HIF) is a heterodimeric transcription factor, stabilized under low oxygen tension to mediate cellular responses, composed of HIFα and the arylhydrocarbon receptor nuclear translocator (ARNT/HIF1β). Studies have shown that the downregulation of GCM1 caused by hypoxia is regulated by HIF. The increased level of GCM1 in *Arnt*-null mouse trophoblast stem (TS) cells induces TS cells differentiated into chorionic trophoblasts and syncytiotrophoblasts (194). Similarly, *Arnt* KO was also shown to partially restore the secretion of hCG in primary human trophoblasts (195). Furthermore, the inhibition of STB differentiation induced by hypoxia is also related to members of the ligand-activated nuclear hormone receptor superfamily, such as peroxisome proliferator-activated receptor gamma (PPARγ). PPARγ is expressed in villous CTBs and is activated during their differentiation into STB (196). It has been reported that *PPARγ*-deficient embryos die at 10.5-11.5 dpc due to placental labyrinth deformation (197). *PPARγ*-null TS cells showed a defect in differentiation into labyrinthine trophoblasts (198). GCM1 reduction in mouse placenta leads to defective STB differentiation and gestational hypertension in later pregnancy, a phenotype resembling PE (100). A previously study showed that the treatment of differentiating TS cells with a PPARγ agonist induced GCM1 expression. Conversely, the overexpression of PPARγ in *PPARγ*-null TS cells promoted both the expression of GCM1 and the formation of multinucleated STB *in vitro* (199). In addition, the failure to form a labyrinth and midgestation lethality were observed in both GCM1 and *PPARγ*-null gestations, suggested that PPARγ may regulate syncytiotrophoblast syncytialization *via* GCM1 (199). In another study, PPARγ agonists resulted in increased secretion of hCG and hPL and reduced expression of the apoptosis-related gene *P53* in primary human trophoblasts in a hypoxic environment (the concentration of oxygen is 1%) (200). Hypoxia can play a critical role in the induction of human IUGR by inhibiting the differentiation of STB *via* GCM1 downregulation. However, there is very little evidence to support the fact that HIF and PPARγ play a role in hypoxia-induced human fetal IUGR. Other studies have shown that hypoxia can lead to an increase in placental oxidative stress and reactive oxygen species (ROS). The latter induces placental dysfunction by increasing the levels of cellular DNA damage, apoptosis, and the peroxidation of both proteins and lipids; these factors can all cause fetal IUGR (201).

## Amino acids

Taurine is the most abundant amino acid in the placenta and is expressed in STB layer. Studies have found that taurine can induce the differentiation and fusion of trophoblasts but does not increase the secretion of hCG (202); these findings demonstrated that the biochemical differentiation and morphological differentiation of trophoblasts are two independent processes (198, 203). Studies of placentas from pregnancies complicated by IUGR have identified a reduction in the activity of the taurine transporter (TauT) and syncytia formation failure; the mechanisms underlying these effects, however, remain unknown (202). However, some researchers have speculated that these effects may be related to the involvement of taurine in transducing intracellular differentiation signals or maintaining intercellular molecular exchange (204, 205). Moreover, STB showed enhanced macropinocytosis induced by mTOR signaling inhibition, which serves as an essential adaptation to amino acid shortages in the placentas from pregnancies complicated by fetal growth restriction patients (206).

## Senescence and syncytialization

Cellular senescence is characterized by cell cycle arrest accompanied by morphological and metabolic changes, including a shift to a proinflammatory phenotype (207). It has a positive effect with regard to limiting injured cell replication, inhibiting tumor growth, and facilitating cell fusion. However, senescence can also induce tumorigenesis as well as a number of age-related pathologies as senescent cells begin to accumulate (208–210). Senescence is caused by oncogene activation, telomere shortening, oxidative stress, and other types of stress leading to DNA damage (211, 212). Interestingly, the fusion of cells mediated by fusogenic proteins can also induce placental senescence, which p21 is activated and the p53 pathway of senescence becomes functional; this activates the p16-pRb (retinoblastoma protein)-dependent pathway, thus inhibiting the proliferation of CTBs (213–215). Cox et al. considered that senescence involves an extension of cell volume, as it provides sufficient space for the continued formation of multinucleated STB; this would also allow the terminally differentiated syncytia to function well and sustain pregnancy (207, 216). Furthermore, senescence can exert an anti-apoptotic effect (217), thus explaining the existence of nonapoptotic yet Caspase-8-positive STB expressing decoy receptor 2 (DCR2), an anti-apoptotic marker of senescence (217, 218).

Some studies reveal that the occurrence IUGR may provide a link to accelerated placental aging, senescence and major obstetric complications, and an arrested release of syncytial knots are observed in the placentas of pregnancies complicated by IUGR (219, 220). Telomerase is an enzymatic complex that completes the replication of telomeres, genetic elements that cap and protect the ends of chromosomes (221). Suppression of telomerase activity and reduced telomere length was found in IUGR placenta with elevated expression of telomere-induced senescence biomarkers, p21, p16 and elongation factor 1 alpha (EF-1α) (219).

## Autophagy and syncytialization

Autophagy protects cells from senescence by degrading and recycling senescence-related components such as misfolded proteins and damaged organelles in a lysosome-embedded manner. Autophagy can be activated under conditions of mild stress and involves two essential organelles: the mitochondria and the endoplasmic reticulum (ER) (222). Calcium can be transferred between these two organelles in a bidirectional manner by virtue of the mitochondria-associated ER membrane (MAM), which is similar to the synapses of the nervous system. The MAM features a large number of calcium transporters and ion channels and is known to play an important role in both oxidative and ER stress (223, 224). Another study showed that an important prerequisite for autophagy was the differentiation-dependent downregulation of p53 (225). Autophagy is a constitutive process, and is activated during villous CTBs syncytialization, which provides energy to cells under situations involving the moderate depletion of nutrients or oxidative stress, and is advantageous in terms of the reorganization of organelles and the degradation of cytoplasmic contents (226–228). A change in autophagy activation in response to chemical treatments or the modulation of Beclin-1 expression was shown to result in a reduction in trophoblastic syncytialization (228). Furthermore, the unfolded protein response (UPR) is activated and protects cells suffering from ER stress during syncytialization, thus inducing autophagy and apoptosis. Consequently, autophagy plays a pivotal role in cell fusion and differentiation. However, increased levels of autophagy have been reported in placentas from pregnancies complicated by IUGR with or without PE; this process provides a nutritional reserve to protect the fetus from acute deprivation (229, 230).

## Conclusion

In this review, we primarily discuss the regulators of trophoblast syncytialization and their aberrant expression in placentas of pregnancies complicated by IUGR. For syncytialization-related proteins, hCG, cadherins, ZO-1, syncytins, GCM1, and 11β-HSD2 are strictly regulated to participate in cell fusion *via* appropriate mechanisms at different fusion stages. Signaling pathways, including the cAMP/PKA, Wnt/β-Catenin, MAPK, PI3K/AKT, JAK/STAT, and TGF-β/SMAD signaling pathways, are involved in coordinating trophoblast syncytialization events and regulating placental function. The DNA methylation of *syncytin-1* and the posttranslational modifications of GCM1 are reported to affect trophoblast syncytialization and associate with fetal IUGR. In addition, metabolic mechanisms, senescence and autophagy are also vital elements involved in regulating the trophoblast syncytialization process.

Thus far, research has shown that the PI3K/AKT, JAK/STAT, and TGF-β/SMAD signaling pathways play key roles in trophoblast syncytialization *in vitro*; however, it is not yet clear how these pathways act on downstream factors and cause syncytialization. Furthermore, little is known about whether the aberrant expression of cAMP/PKA, Wnt/β-Catenin, MAPK, PI3K/AKT, JAK/STAT, and TGF-β/SMAD signaling pathways leads to IUGR by affecting other biological behaviors of trophoblasts, as the etiologies of IUGR are diverse. Thus, clarifying whether these signaling pathways participate in the pathology of IUGR caused by inadequate trophoblast syncytialization and how they act on syncytialization-related molecules is of great significance to clarify the mechanism underlying IUGR development.

This review provides us with not only a better understanding of the pathogenesis of placental dysfunction caused by insufficient trophoblast syncytialization, but also new ideas and insights to select a comprehensive approach to therapy and prevent fetal IUGR occurrence.

## Author contributions

HJZ wrote the article. HJZ, CZ, PW, and WY made the figures and drafted the article. HYZ and SZ critically reviewed the article. All authors contributed to the article and approved the submitted version.
